# Identification of Immune-Related Gene Signature and Prediction of CeRNA Network in Active Ulcerative Colitis

**DOI:** 10.3389/fimmu.2022.855645

**Published:** 2022-03-22

**Authors:** Mengmeng Xu, Ying Kong, Nannan Chen, Wenlong Peng, Ruidong Zi, Manman Jiang, Jinfeng Zhu, Yuting Wang, Jicheng Yue, Jinrong Lv, Yuanyuan Zeng, Y. Eugene Chin

**Affiliations:** ^1^ Institutes of Biology and Medical Sciences, Soochow University, Suzhou, China; ^2^ Department of Pathology, The Second Affiliated Hospital of Soochow University, Suzhou, China; ^3^ Department of Respiratory Medicine, The First Affiliated Hospital of Soochow University, Suzhou, China

**Keywords:** active UC, GEO dataset, integrated analysis, AGP, gene signatures, ceRNA network

## Abstract

**Background:**

Ulcerative colitis (UC) is an inflammatory disease of the intestinal mucosa, and its incidence is steadily increasing worldwide. Intestinal immune dysfunction has been identified as a central event in UC pathogenesis. However, the underlying mechanisms that regulate dysfunctional immune cells and inflammatory phenotype remain to be fully elucidated.

**Methods:**

Transcriptome profiling of intestinal mucosa biopsies were downloaded from the GEO database. Robust Rank Aggregation (RRA) analysis was performed to identify statistically changed genes and differentially expressed genes (DEGs). Gene Set Enrichment Analysis (GSEA), Gene Ontology (GO), and Kyoto Encyclopedia of Genes and Genomes (KEGG) were used to explore potential biological mechanisms. CIBERSORT was used to evaluate the proportion of 22 immune cells in biopsies. Weighted co-expression network analysis (WGCNA) was used to determine key module-related clinical traits. Protein-Protein Interaction (PPI) network and Cytoscape were performed to explore protein interaction network and screen hub genes. We used a validation cohort and colitis mouse model to validate hub genes. Several online websites were used to predict competing endogenous RNA (ceRNA) network.

**Results:**

RRA integrated analysis revealed 1838 statistically changed genes from four training cohorts (adj. *p-*value < 0.05). GSEA showed that statistically changed genes were enriched in the innate immune system. CIBERSORT analysis uncovered an increase in activated dendritic cells (DCs) and M1 macrophages. The red module of WGCNA was considered the most critical module related to active UC. Based on the results of the PPI network and Cytoscape analyses, we identified six critical genes and transcription factor NF-κB. RT-PCR revealed that andrographolide (AGP) significantly inhibited the expression of hub genes. Finally, we identified XIST and three miRNAs (miR-9-5p, miR-129-5p, and miR-340-5p) as therapeutic targets.

**Conclusions:**

Our integrated analysis identified four hub genes (*CXCL1*, *IL1B*, *MMP1*, and *MMP10*) regulated by NF-κB. We further revealed that AGP decreased the expression of hub genes by inhibiting NF-κB activation. Lastly, we predicted the involvement of ceRNA network in the regulation of NF-κB expression. Collectively, our results provide valuable information in understanding the molecular mechanisms of active UC. Furthermore, we predict the use of AGP and small RNA combination for the treatment of UC.

## Introduction

Ulcerative colitis (UC) is a type of relapsing/remitting inflammatory bowel disease characterized by clinical symptoms of abdominal cramping, passage of pus, mucus, or both, and bloody diarrhea (1). UC imposes a major health burden globally, owing to its high incidence in developed countries and the increasing prevalence in developing countries, occurring across all ages (2, 3). The pathophysiology of UC is multifaceted and not completely understood. Environmental factors, genetic susceptibility, intestinal epithelium barrier defects, and dysfunctional immune responses have all been suggested to contribute to UC pathogenesis (4, 5).

In general, UC patients often experience two phases of the disease, namely the active phase, during which the symptoms are present, and the remission phase, characterized by the absence of symptoms (6). UC is considered a progressive disease, giving rise to a variety of intestinal disorders, including colorectal cancer, thus compromising the patient’s quality of life (4, 7). Currently, UC treatment goals include early remission and long-term maintenance to prevent relapse. Oral and local 5-*aminosalicylic acid* (5-ASA) formulations are useful for achieving remission in patients with mildly to moderately active UC. Successful 5-ASA treatment in UC patients reduced the risk of UC-associated colorectal cancer (8). Longitudinal analysis revealed that vedolizumab, a monoclonal antibody directed against the integrin heterodimer α4β7, induced the recovery of intestinal mucosal injury owing to its anti-inflammatory effects (9). Andrographolide (AGP) is a natural product extracted from traditional Chinese herbs and has been shown to alleviate clinical UC symptoms with minor side effects (10). AGP was reported to block the NF-κB signaling pathway in macrophages (11, 12) and regulate neutrophil activation, apoptosis, and extracellular trap formation (13, 14). CX-10, an AGP derivative, reduced dextran sulfate sodium (DSS)‐induced tissue damage in mice by blocking NF-κB and MAPK signaling (15).

Under normal intestinal barrier conditions, only few luminal antigens and microbiota find their way into the lamina propria. However, when damage compromises barrier integrity and tolerance mechanisms fail, a complex population of immune cells within the intestinal lamina propria are exposed to the invading luminal antigens, which leads to the excessive infiltration of immune cells, as well as chemokine and cytokine production, which further exacerbate inflammation. Infiltrating cell types include neutrophils (16), dendritic cells (17), innate lymphoid cells (18), natural killer T cells (19), macrophages (20), and T cells (21, 22). Activated immune cells communicate mutually *via* direct or indirect contact through the secretion of cytokines, such as tumor necrosis factor (TNF), interferon gamma (IFNγ), interleukin 1β (IL1β), IL-6, and IL-23, as well as T helper (Th) 17 cell-associated cytokines. Chemokines form another large family of inflammatory factors that regulate leukocyte trafficking and activation (23, 24).

The complex intestinal microenvironment complicates UC diagnosis and treatment. Therefore, uncovering the underlying etiology of UC is necessary for the development of curative treatment. Recently, high-throughput sequencing methods have provided unprecedented insight for the study of disease mechanisms and biomarker identification (25, 26). In the present study, we screened key signature genes using robust rank aggregation (RRA), weighted co-expression network analysis (WGCNA), and Cytoscape software. We then validated signature genes in patient datasets and colitis mouse model. Finally, we constructed a lncRNA–miRNA–transcription factor (TF) interaction network to provide a preliminary plan for combination therapy in active UC.

## Materials and Methods

### Datasets and Sample Selection

We systematically searched for microarray studies from public GEO datasets using the following terms: “mucosal” and “ulcerative colitis”. The screening criteria were as follows: ① expression profiling by array; ② *Homo sapiens*; ③ dataset containing more than five active UC samples and healthy controls. Finally, five GEO datasets (GSE16879, GSE75214, GSE48958, GSE87473, and GSE92415) were included (27–30) (**Table 1**). We downloaded series matrix file(s) from the GEO website and corresponding annotation documents from GEO or Bioconductor.

**Table 1:** Microarray information.

GEO ID	Platform	Participants	Tissues	Attribute
GSE16879	GPL570	24 active UC and 12 controls	Mucosal	Training set
GSE48958	GPL6244	7 active UC and 8 controls	Mucosal	Training set
GSE75214	GPL6244	74 active UC and 22 controls	Mucosal	Training set
GSE87473	GPL13158	106 active UC and 21 controls	Mucosal	Training set
GSE92415	GPL13158	87 active UC and 21 controls	Mucosal	Validation set

### RRA Analysis

RRA analysis was used to integrate gene expression data from different datasets in an unbiased manner using a comprehensive ranking list algorithm (31). We combined the “limma” and “RobustRankAggreg” packages in *R* to obtain statistically changed genes (adj. *p*-value < 0.05) and differentially expressed genes (DEGs; adj. *p*-value < 0.05 and |logFC| > 1.5).

### Biological Function and Pathway Enrichment Analyses

Gene Set Enrichment Analysis (GSEA), Gene Ontology (GO) analysis, and Kyoto Encyclopedia of Genes and Genomes (KEGG) pathway enrichment analysis were conducted using the “ClusterProfiler” *R* package (32). The reference gene list c2.cp.reactome.v7.0.symbols.gmt was downloaded from Molecular Signature Database (MsigDB) (33). GO analysis included the biological process (BP), cellular component (CC), and molecular function (MF) categories. FDR < 0.05 was considered significant.

### Evaluation of Tissue-Infiltrating Immune Cells

The CIBERSORT deconvolution algorithm was employed to analyze the cellular composition of the tissues based on gene expression profiles, according to the known reference set LM22 (leukocyte signature matrix) (34). The permutation (perm) was set at 1000 to obtain more stable results.

### WGCNA

WGCNA can be used as a data exploratory tool or screening method to identify key gene modules using an unsupervised clustering without priori defined gene sets (35). In our study, a total of 15162 genes (top 75% according to variation) were analyzed using the “WGCNA” *R* package. Scale-free network features were constructed when the power of β was equal to 12 (R^2^ = 0.85). Dynamic tree cut algorithm was used to aggregate genes with similar expression profiles into the same module. Gene clusters in the module most related to the active UC traits were considered candidates for further validation.

### PPI Network Analysis

STRING database (https://cn.string-db.org/) is a functional protein association network, assembling all known and predicted proteins (36). Multiple protein names were put into the list box, with one name per line. PPI network interactions file with medium confidence scores ≥ 0.4 was downloaded. Cytoscape software (version 3.7.2), a general-purpose, open-source software platform for network biology analysis and visualization (37). Molecular complex detection (MCODE) (K-core = 2, degree cutoff = 2, max depth = 100, and node score cutoff = 0.2) and cytohubba-MCC plugins with default parameters were used to explore important gene clusters and hub genes (38, 39). The iRegulon (Version: 1.3) plugin was used to screen key TF with the default cutoff criteria (40).

### Correction Analysis

We used the *R* “corrplot” package to display a correlation matrix. We download the organized gene list of “Immunomodulator” from TISIDB database (http://cis.hku.hk/TISIDB/index.php), which is an online integrated repository portal for tumor-immune system interactions (41). *p*-value < 0.05 was considered to be statistically significant.

### Animal Model of Colitis

Animal experiments were approved by the Animal Ethics and Experimentation Committee of Soochow University and carried out according to the Guide for the Care and Use of Laboratory Animals. AGP or DMSO diluted in PBS was intraperitoneally injected into mice at a dose of 25 mg/kg on alternate days, with the first dose administered one day before DSS administration. The experimental colitis model was induced in 10-week-old *C57BL/6* male mice by administering 3.5% DSS (MW: 36,000–50,000 Da; Yeasen Biotechnology Co., Ltd., Shanghai, China, 60316ES76) for seven days, followed by administration of normal water for three days. Mice were sacrificed on the tenth day, and colon tissues were obtained for hematoxylin and eosin (H&E) staining, immunofluorescence (IF) staining, and RT-PCR analysis.

### H&E and IF Staining

Colon tissues were fixed with 4% paraformaldehyde (PFA) overnight and were then embedded in paraffin. Colonic sections of 5 μm were obtained and laid flat on a glass slide for H&E or IF staining. For IF staining, the tissue slices were permeabilized with 0.1% Triton X-100 for 15 min, blocked with 1% BSA for 1 h at 37°C, and then incubated with antibodies against NF-κB (Cell Signaling Technology, #8242, 1:500) and IκBα (Cell Signaling Technology, #4812, 1:500) at 4°C overnight. The next day, tissue slices were incubated with an Alexa Fluor^®^ 488-conjugated goat anti-rabbit IgG antibody (Life Technologies, A11088) for 1 h at room temperature. The nuclei were stained with Hoechst 33342 (1:10000 dilution, Beyotime) for 10 min prior to imaging. Representative cells were selected and photographed.

### RNA Extraction and RT-PCR

RNA was extracted from the intestinal tissues using TRIzol reagent (Invitrogen, 15596018) and reverse-transcribed to cDNA using the Hifair^®^ II 1st Strand cDNA Synthesis Kit (Yeasen Biotechnology, 11119ES60). Primer sequences were obtained from PrimerBank (https://pga.mgh.harvard.edu/primerbank/) and synthesized at GENEWIZ Biotech Co. Ltd. (Suzhou, China) (**Table 2**). RT-PCR was performed using the Hieff^®^ qPCR SYBR Green Master Mix (High Rox Plus; Yeasen Biotechnology, 11203ES03) on an ABI Applied Biosystems. PCR amplification was conducted in triplicate for each sample, and the expression of target genes was normalized to *GAPDH*. Relative expression was determined using the 2^-ΔΔCt^ method.

**Table 2:** Primers for RT-PCR.

Gene	Primer	Sequence (5’ -> 3’)
*CXCL1*	Forward	ACTGCACCCAAACCGAAGTC
	Reverse	TGGGGACACCTTTTAGCATCTT
*IL-1B*	Forward	GCAACTGTTCCTGAACTCAACT
	Reverse	ATCTTTTGGGGTCCGTCAACT
*MMP1*	Forward	CTTCTTCTTGTTGAGCTGGACTC
	Reverse	CTGTGGAGGTCACTGTAGACT
*MMP3*	Forward	ACATGGAGACTTTGTCCCTTTTG
	Reverse	TTGGCTGAGTGGTAGAGTCCC
*MMP7*	Forward	CTTACCTCGGATCGTAGTGGA
	Reverse	CCCCAACTAACCCTCTTGAAGT
*MMP10*	Forward	GAGCCACTAGCCATCCTGG
	Reverse	CTGAGCAAGATCCATGCTTGG
*TIMP1*	Forward	CGAGACCACCTTATACCAGCG
	Reverse	ATGACTGGGGTGTAGGCGTA
*PLAU*	Forward	ATGGAAATGGTGACTCTTACCGA
	Reverse	TGGGCATTGTAGGGTTTCTGA
GAPDH	Forward	AGGTCGGTGTGAACGGATTTG
	Reverse	TGTAGACCATGTAGTTGAGGTCA

### ceRNA Network Construction

TargetScan Human 7.2 (http://www.targetscan.org/vert_72/), miRDB (http://mirdb.org/), microT v5 (http://diana.imis.athena-innovation.gr/DianaTools/index.php?r=microT_CDS/index), and miRWalk 3.0 (http://mirwalk.umm.uni-heidelberg.de/) with default parameters were used to predict the miRNAs (42–45). StarBase (v2.0) (https://starbase.sysu.edu.cn/starbase2/index.php) was used to predict miRNA-lncRNA interactions with very high stringency (≥5) (46). The interaction networks were constructed and visualized using Cytoscape software.

### Statistical Analyses

The *R* “ggpubr” package was used to perform statistical analyses, and the *R* “ggplot2” package was used to generate images. Differences between two groups were assessed using the Student’s *t*-test. All data are shown as mean ± standard error of the mean (SEM). GraphPad Prism 7.0 (GraphPad Software, Inc., La Jolla, CA, USA) was used for statistical analysis and image construction. Adobe Illustrator (AI) CS6 software was used to edit the figures.

## Results

### GSEA Revealed the Involvement of the Innate Immune system

**Figure 1:**
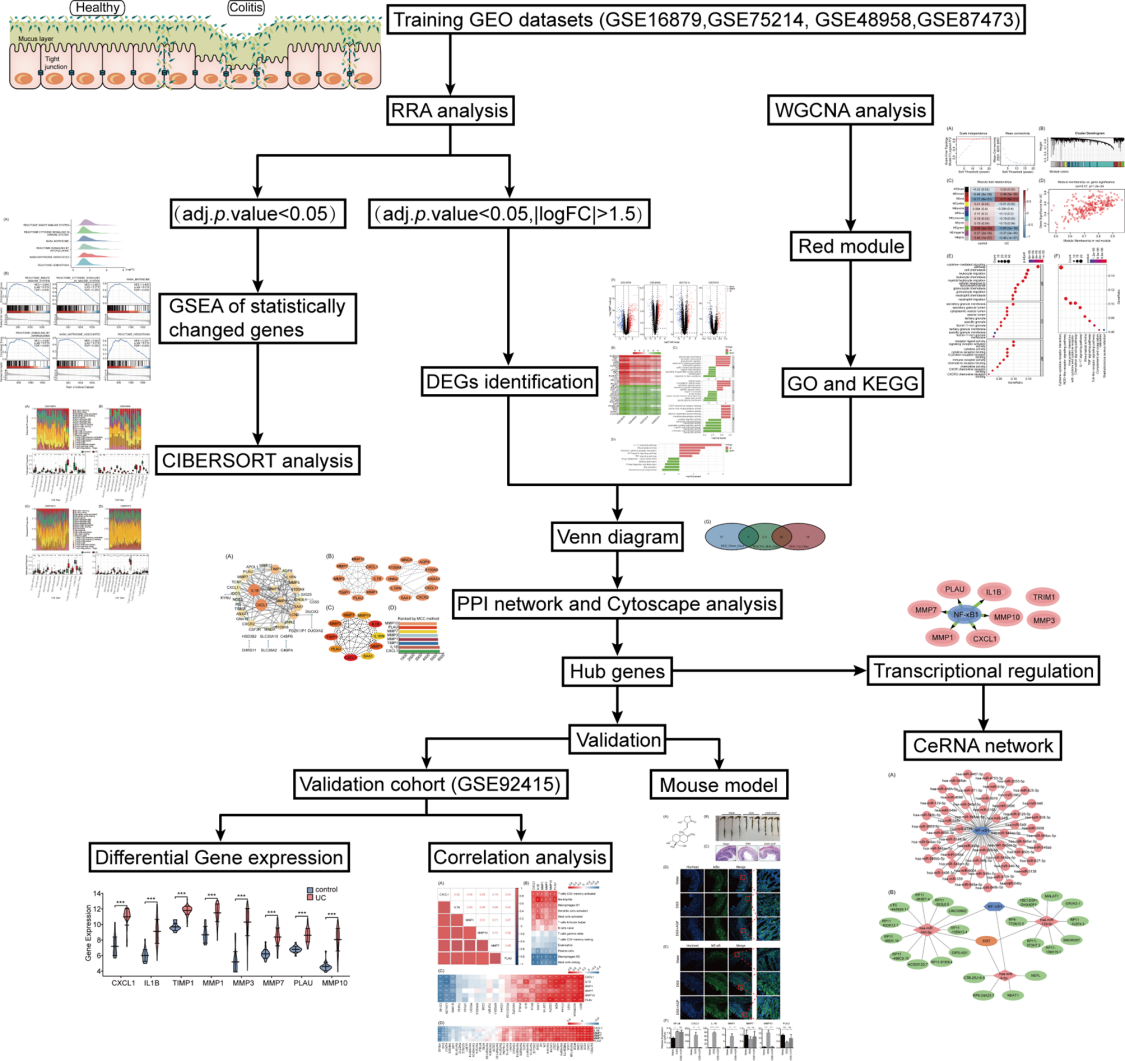
Flowchart of the study.

**Figure 2:**
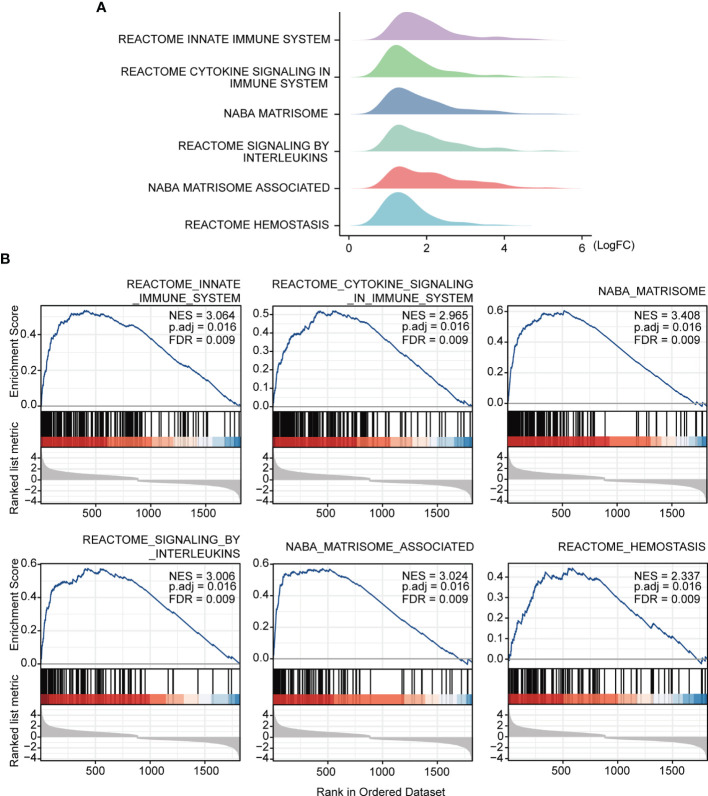
GSEA annotation of gene sets. **(A)** Ridge plot show gene expression distribution in the immune-related annotated gene set. **(B)** Gene set enriched in the innate immunity system (FDR = 0.009, NES = 3.064, adj. p-value < 0.05), gene set enriched in cytokine signaling in immune system (FDR = 0.009, NES =2.965, adj. p-value < 0.05), gene set enriched in the NABA matrisome (FDR = 0.009, NES = 3.408, adj. p-value < 0.05), gene set enriched in signaling by interleukins (FDR = 0.009, NES = 3.006, adj. p-value < 0.05), gene set enriched in NABA matrisome associated (FDR = 0.009, NES = 3.024, adj. p-value < 0.05), gene set enriched in hemostasis (FDR = 0.009, NES = 2.337, adj. p-value < 0.05). Screening criteria for significant gene sets included adj. *p-*value < 0.05 and FDR < 0.25. NES: normalized enrichment score.

The study workflow is presented in **Figure 1**. GSE16879, GSE48958, GSE75214, and GSE87473 expression data were processed and normalized. Boxplots show the normalized gene expression profiles, and principal component analysis (PCA) scatter plots show significant differences between active UC samples and normal controls (**Supplementary Figures 1A–D**). We performed RRA analysis to determine statistically changed genes in different datasets and then subjected them to GSEA. RRA analysis revealed 1838 statistically changed genes (adj. *p*-value < 0.05). GSEA indicated that the top annotated collection of genes was enriched in innate immune system, indicating that innate immune responses may play an important role during active UC (**Figures 2A, B**).

### Immune Landscape of the Training GEO Datasets

**Figure 3:**
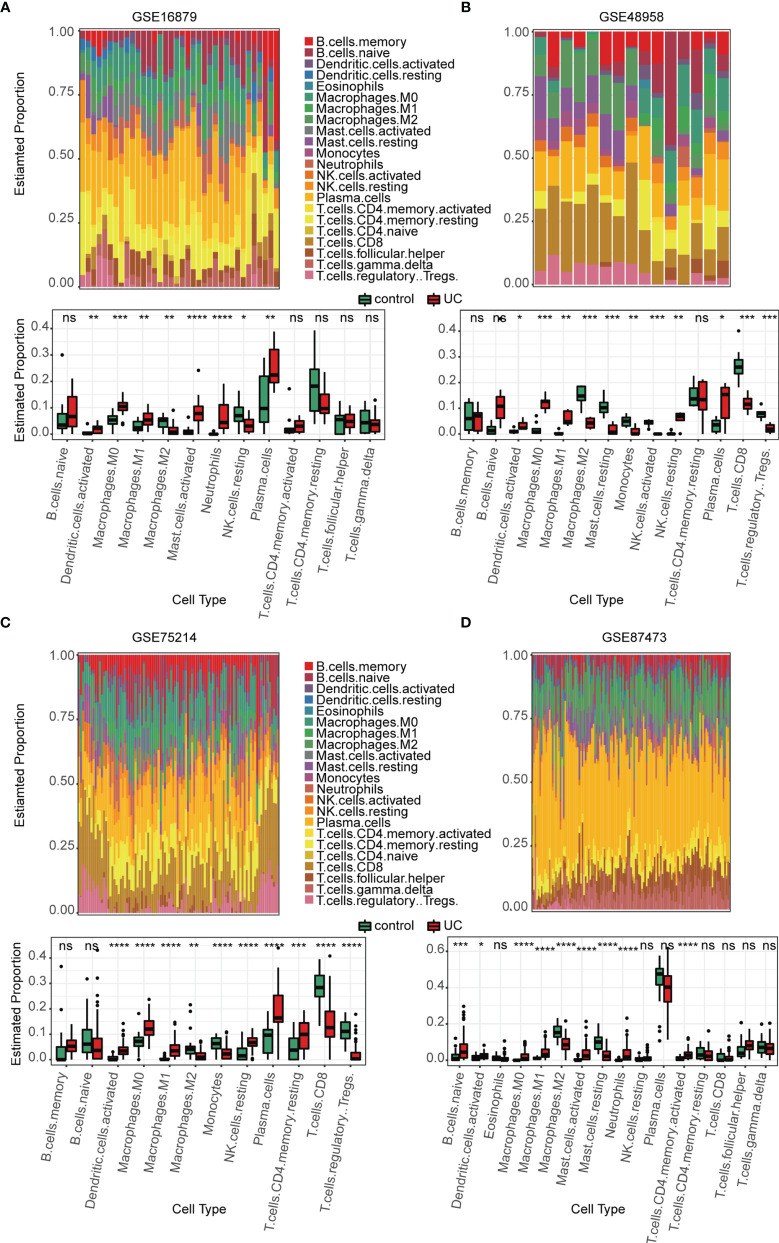
Estimation of infiltrating immune cell types in the 4 training GEO cohorts *via* CIBERSORT. **(A–D)** Stacked barplots show the relative composition of 22 immune cell subsets in four cohorts, and the boxplots show that activated DCs and macrophages (M0 and M1) were significantly increased in the active UC group. Data were assessed via the method of Benjamini and Hochberg (BH). * adj. *p-*value < 0.05, ** adj. *p-*value < 0.01, *** adj. *p-*value < 0.001, **** adj. *p-*value <0.0001, ns, no significance.

Several immune-related gene sets were enriched in the active UC group as compared with that in the normal group, especially with regards to the innate immune system. We explored the immune landscape in training datasets using the CIBERSORT algorithm. The abundance of 22 immune cell types is shown using stacked bar plots (**Figures 3A–D**). Cell types were filtered if not present in half of the samples, and their relative proportions are shown in the boxplots (**Figures 3A–D**). The results revealed that the active UC tissue was infiltrated by a higher fraction of activated DCs and macrophages (M0 and M1) and a lower fraction of M2 macrophages in all training datasets (FDR < 0.05), whereas other immune cells exhibited heterogeneity (**Figures 3A–D**). These results indicated that the inflammatory microenvironment reshapes the proportion and distribution of immune cells.

### Identification of DEGs and Functional Enrichment Analysis

**Figure 4:**
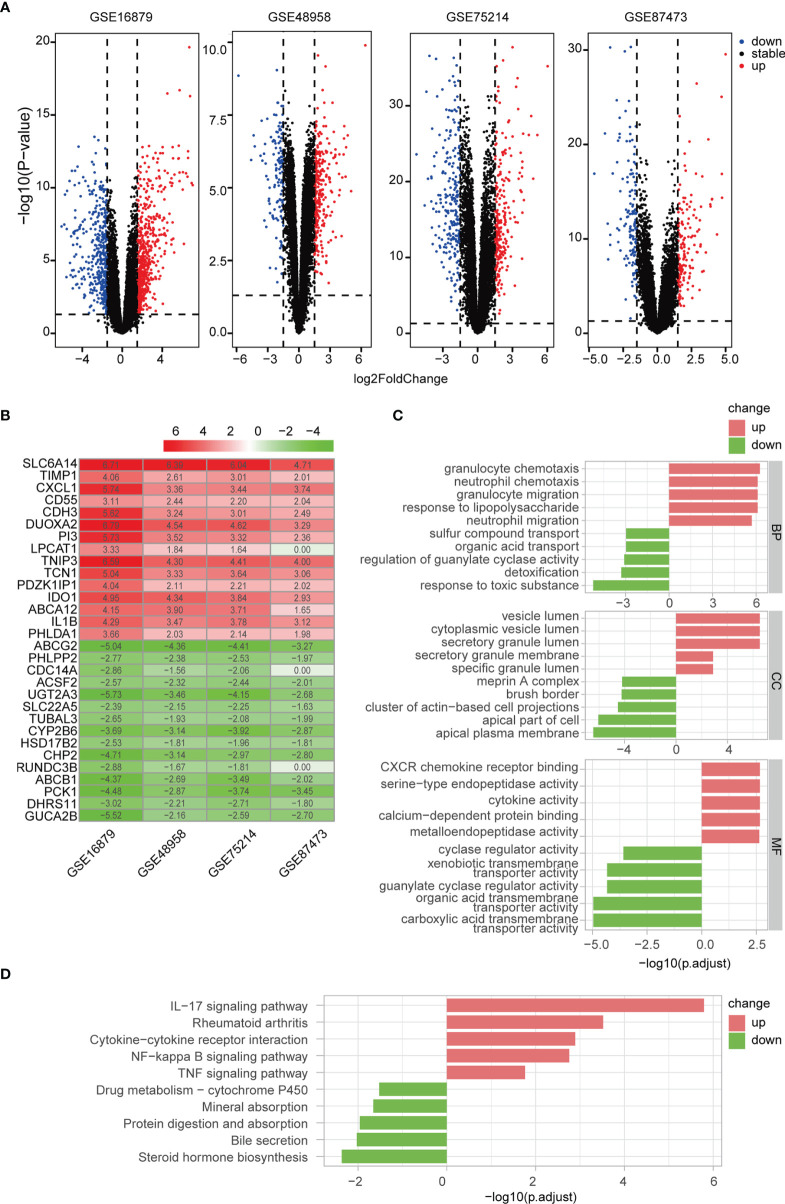
Differentially regulated genes (DEGs) were identified *via* RRA analysis. **(A)** Volcano plots show DEGs in active UC and control samples from the four GEO datasets. **(B)** Heatmap of the top 15 up- and down-regulated DEGs from the integrated analysis. **(C)** GO functional enrichment analysis, including BP, CC, and MF, revealed the underlying functions of up- and down-regulated DEGs. **(D)** KEGG revealed the top ten pathways enriched in up- and down-regulated DEGs.

We identified 1072, 431, 490, and 303 DEGs between active UC patients and normal subjects in the GSE16879, GSE48958, GSE75214, and GSE87473 datasets, respectively (**Figure 4A**). Subsequently, RRA analysis integrated the four cohorts, revealing 64 up-regulated and 46 down-regulated DEGs. The top 15 upregulated and downregulated genes are shown using a heatmap (**Figure 4B**). Furthermore, GO and KEGG functional enrichment analyses were performed to determine the biological features of these 110 robust DEGs. GO functional enrichment analysis revealed 487 up-regulated and 221 down-regulated terms (FDR< 0.05) across BP, CC, and MF categories. DEGs were markedly enriched in granulocyte and neutrophil chemotaxis and migration in the BP category. In the CC category, genes were mainly enriched in vesicle and secretory granule terms. Enriched MF terms included cytokine activity and metallopeptidase activity (**Figure 4C**). KEGG pathway analysis revealed 16 up-regulated and 5 down-regulated items (FDR < 0.05), which included the IL-17 and NF-κB signaling pathways (**Figure 4D**). These results indicated that pro-inflammatory factors and pathways were enriched in active UC patients.

### Co-Expression Modules and Functional Enrichment Analysis

**Figure 5:**
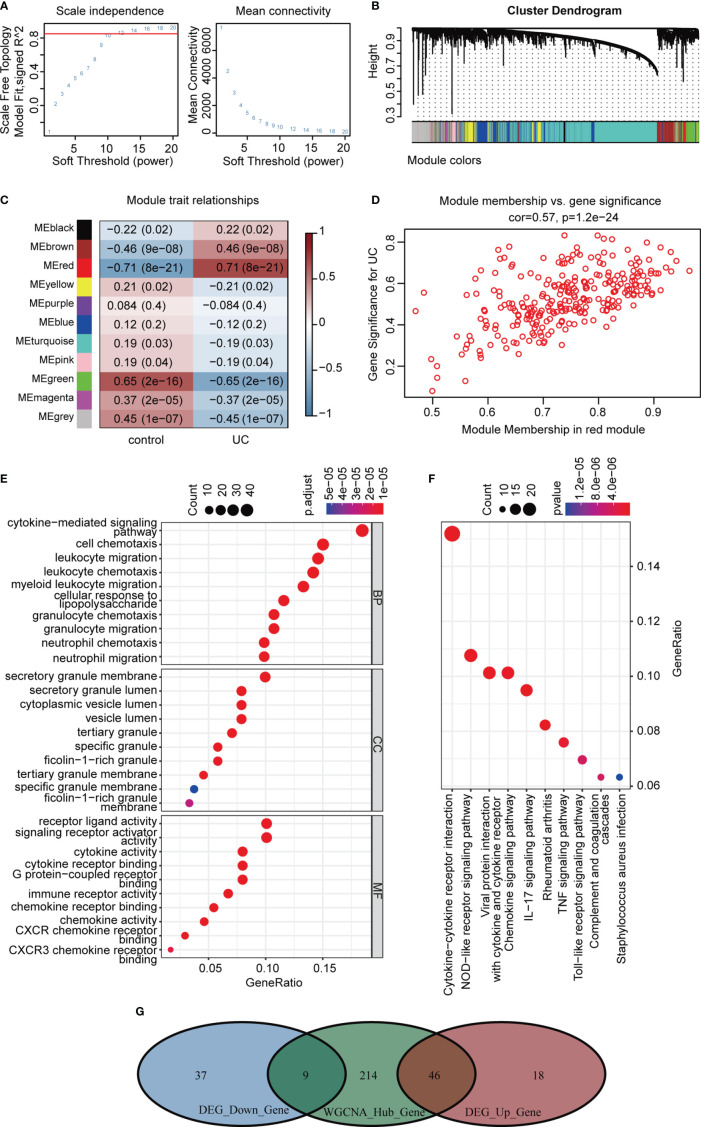
WGCNA analysis of GSE87473. **(A)** Soft threshold power screening and scale-free network construction. **(B)** The hierarchical clustering tree shows the network and the 11 identified modules. **(C)** Heatmap plot shows the adjacencies in the eigengene network. Correlation of each module with active UC. **(D)** A scatterplot of gene significance (GS) versus module membership (MM) in the red module. **(E, F)** GO and KEGG enrichment analysis of the red module. **(G)** Venn diagram of the overlapping genes.

Correlation networks are used for identifying clusters of highly correlated genes across microarray samples. We employed WGCNA to construct and analyze active UC-associated networks. We analyzed GSE87473 microarray datasets, containing 106 active UC and 21 normal control samples, and the optimal β value to match approximate scale-free topology criterion compared to other datasets. The adjacency matrix was constructed based on the criterion of gene distribution conformed to a scale-free network when setting the soft-threshold power of β = 12 (R^2^ = 0.85), retaining high connectivity information (**Figure 5A**). In this study, gene clusters were conducted using the hierarchical clustering method, and 11 consensus co-expression modules were identified (**Figure 5B**). The heatmap shows the association between each module and clinical traits (**Figure 5C**). The correlation analysis of module membership (MM) and gene significance (GS) indicated that the selected module genes exhibited good correlation with the red MM (R = 0.57, P = 1.2e-24, **Figure 5D**), implying that genes in the red module were highly correlated with active UC. Genes within the red module were selected, and their biological function was inferred using GO and KEGG analyses (**Figures 5E, F**). Enrichment analyses yielded immune-related terms and pathways. A total of 55 overlapping DEGs were obtained *via* RRA analysis and WGCNA (**Figure 5G**).

### PPI Network Construction and Key TF Identification

**Figure 6:**
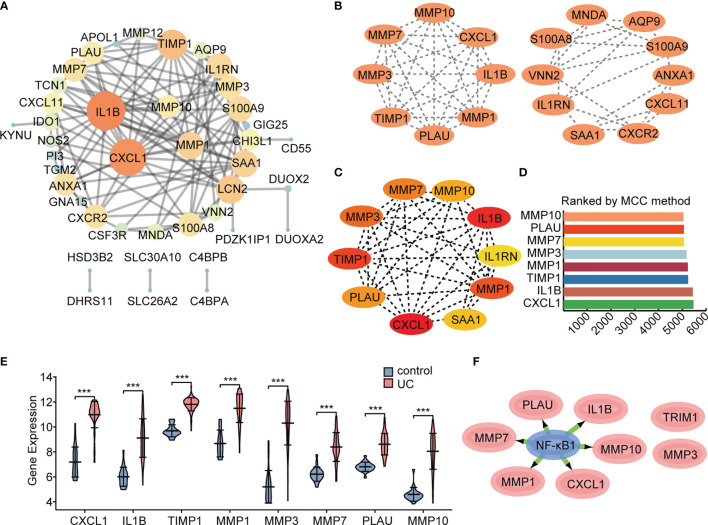
Protein interaction network. The network was constructed and visualized using Cytoscape software. **(A)** PPI network. The nodes represent proteins, the edges represent their interaction. **(B)** MCODE sub-network, including cluster 1 and cluster 2. **(C, D)** Cytohubba-MCC was used to identify hub genes in the network. **(E)** Differential gene expression analysis in GSE92415 cohort. **(F)** The master regulator predicted by the iRegulon tool is highlighted in blue, and target genes are in pink. ***P < 0.001.

An interaction network between proteins encoded by the 55 DEGs was constructed using the STRING database (**Figure 6A**). The interaction network comprised 49 nodes and 88 edges, visualized using the Cytoscape software. The MCODE plugin was used to identify gene cluster modules. According to the filter criteria, two cluster modules were identified. Cluster 1 had the higher score (score: 8.000, 8 nodes and 28 edges), followed by cluster 2 (score: 5.111, 10 nodes and 23 edges; **Figure 6B**). Next, we used the cytoHubba-MCC plugin to identify hub genes and obtained eight hub genes when setting score >5000 (**Figures 6C, D**), consistent with cluster 1 in MCODE. We performed differential expression analysis of the eight hub genes in the validation cohort GSE92415. Consistent with our prediction, mRNA expression levels of the eight specifically expressed hub genes were significantly upregulated in active UC samples compared to those in the control samples (**Figure 6E**). Gene expression is spatiotemporally regulated *via* networks, which consist of interactions between TFs and their direct target genes, influencing development, homeostasis, and pathogenesis. Eight hub genes correlated with active UC traits were tested for TF binding motifs using the iRegulon plugin. The results indicated that six genes, excluding *TIMP1* and *MMP3*, were regulated by NF-κB1 (**Figure 6F**).

### Hub Genes Correlation Analysis

**Figure 7:**
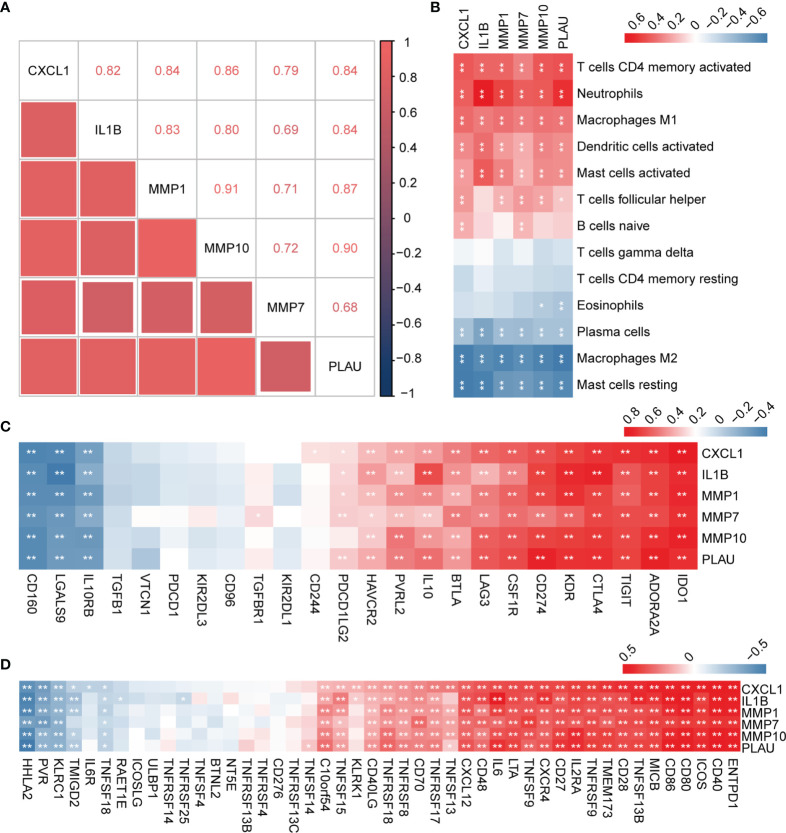
Correlation analysis between hub genes with immune cell types and factors. **(A)** Correlation analysis of the six hub genes. **(B)** Correlation analysis of hub genes and different immune cell types. **(C, D)** Correlation analysis of hub genes with immunosuppressive and immunostimulatory factors. *P < 0.05, **P < 0.01.

In order to explore the expression patterns of hub genes, we performed correlation analysis of the expression data from the GSE92415 validation cohort. Positive correlation was observed between the hub genes (**Figure 7A**). We further explored the relationship between hub genes and immune cells, and the hub genes were significantly associated with pro-inflammatory cell types, such as neutrophils, M1 macrophages, and activated DCs. Hub genes showed a negative correlation with M2 macrophages and plasma cells (**Figure 7B**). We then analyzed the correlation between hub genes and different immune regulators, including immunosuppressive and immunostimulatory factors (**Figures 7C, D**). These analyses verified that hub genes were closely involved in the regulation of immune cell infiltration and inmmunoregulators, thus modulating the immune microenvironment.

### Inhibition of NF-κB Activity Suppressed the Expression of Hub Genes and Alleviated Colitis

**Figure 8:**
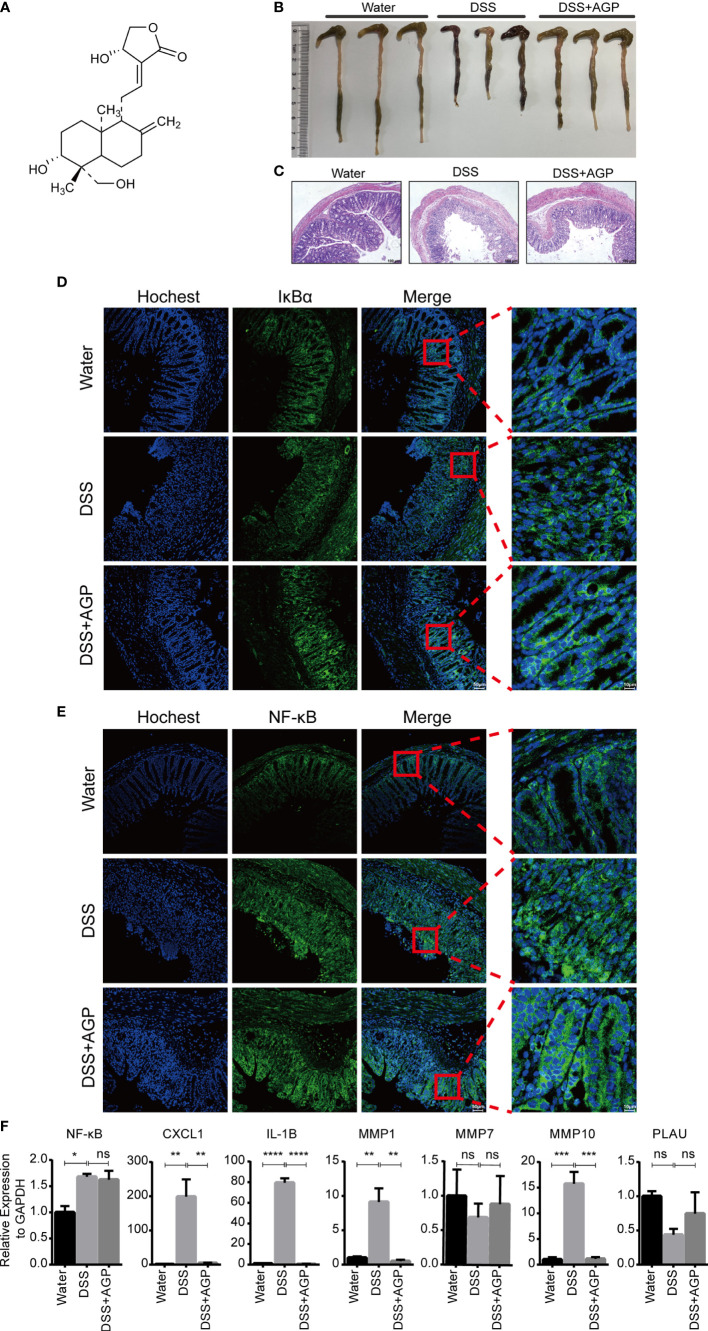
AGP alleviated DSS-induced colitis. **(A)** The structure of AGP. **(B)** Colon length of mice during 3.5% DSS challenge. **(C)** Representative H&E staining of the colon at day 10 of colitis induction. **(D, E)** AGP prevents nuclear translocation of NF-κB without affecting IκBα. **(F)** RT-PCR analysis. *P < 0.05, **P < 0.01, ***P < 0.001, ****P < 0.0001, and ns, no significance (Student’s t-test). Error bars represent the SEM.

To further validate the important role of hub genes in active UC, we employed the DSS-induced mouse colitis model. Studies have shown AGP and its derivates could ameliorate active UC symptoms and promote barrier integrity through the inhibition of NF-κB signaling (15, 47). NF-κB represents a family of TFs containing five members, with the NF-κB p65–p50 (NF-κB1) heterodimer being most widely studied. Mice were intraperitoneally injected with DMSO or AGP during the period of DSS administration (**Figure 8A**). AGP-treated mice exhibited milder colon blockage than that in the control mice (**Figure 8B**). Histologic analysis strengthened the protective effect of AGP against colitis, with less intestinal epithelial damage and more preserved goblet cells (**Figure 8C**). IF staining revealed that AGP attenuates inflammation by preventing NF-κB activation without affecting IκBα expression (**Figures 8D, E**). We also detected the expression levels of NF-κB and hub genes using RT-PCR. NF-κB activity was increased in the DSS group, whereas there was no significantly recused in the DSS-AGP treatment group. *CXCL1*, *IL1B*, *MMP1*, and *MMP10* were significantly upregulated in the DSS group; however, the effect was rescued by AGP treatment. No significant changes were observed in *MMP7* and *PLAU* expression (**Figure 8F**). Our results revealed four genes implicated in the progression of active UC, providing insight into the therapeutic mechanism of the traditional herb AGP.

### CeRNA Network Construction

**Figure 9:**
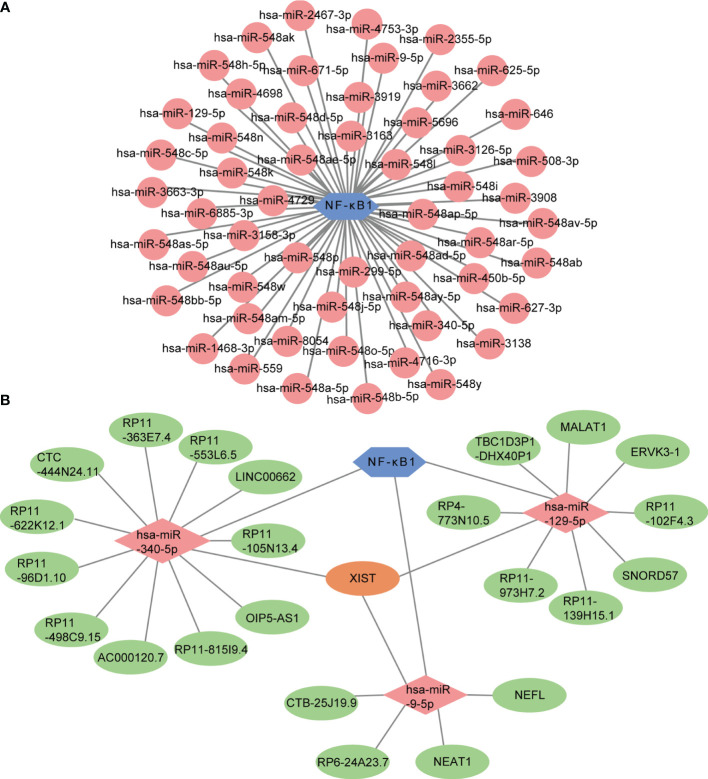
Prediction of miRNA-mRNA network and ceRNA network. **(A)** The miRNA-mRNA network included NF-κB (blue hexagon) and 55 regulatory miRNAs (pink circles). **(B)** The lncRNA-miRNA-mRNA network was constructed, including three lncRNA-miRNA subnetworks and XIST showed as the crossover point. The blue hexagon node, the pink diamond nodes, and the pale-green ellipse nodes represent the mRNA and miRNA, respectively. Apricot ellipse nodes represent crosstalk *via* XIST.

MicroRNAs are widely studied refer to epigenetic regulators that have shown tremendous potential as therapeutic targets for various human diseases. Four online miRNA databases were used to predict the miRNAs regulating NF-κB activity. Interactions indicated by all three miRNA databases were selected. We obtained 55 regulatory miRNAs of NF-κB and constructed a network using the Cytoscape software (**Figure 9A**). The ceRNA network has attracted much attention in recent years and plays an important role in regulating gene expression. The ceRNA network was constructed with miRNA as a bridge to establish the relationship between target gene mRNA and lncRNA by combining miRNA response elements (MREs). We further explored the lncRNAs that interacted with regulator miRNAs using the StarBase 2.0 database and constructed three lncRNA–miRNA subnetworks (**Figure 9B**). Interestingly, we found that the lncRNA XIST mediated crosstalk between the three miRNAs. Thus, we speculated that the ceRNA network might play an important role in active UC by regulating NF-κB expression.

## Discussion

Although there have been substantial advances in understanding the immunopathogenesis of UC, critical questions remain to be answered. The underlying etiology and triggers of UC are unclear. In the past few decades, most studies have focused on the role of abnormal adaptive immune responses in the pathogenesis of UC. Advances arising from genome-wide association studies and immunological studies have recently moved the focus of UC pathogenesis on to mucosal innate immune responses (48, 49). In our study, GSEA of 211 samples from patients with active UC and 63 from heathy controls indicated that several immune response-related pathways were involved in UC, especially those of innate immunity. CIBERSORT analysis revealed that activated DC and M1 macrophages were significantly increased in patient colon tissues. Comprehensive bioinformatics analysis using RRA, WGCNA, and Cytoscape, as well as *in vivo* validation using a classic mouse model of colitis revealed *CXCL1*, *IL1B*, *MMP1*, and *MMP10* as signature genes of active UC. Furthermore, a central role of the TF NF-κB in the regulation of active UC was determined. We predicted a ceRNA network of the XIST–miR-9-5p/miR-129-5p/miR-340-5p–NF-κB axis in the regulation of NF-κB expression, providing neww avenues for combination targeted therapy in active UC.

According to the modified Riley score (50), histologic UC activity is defined as the presence of epithelial infiltration by neutrophils, crypt abscesses, erosions, or ulceration. Neutrophil infiltration was confirmed as an early yet central event in UC (51), persisting in parallel to inflammation. Disruption of intestinal macrophage homeostasis contributed to neutrophil infiltration. Macrophages are classified into classically activated M1 or alternatively activated M2 macrophages, and their “plasticity” is based on cytokine secretory patterns and pro-inflammatory versus immunoregulatory activity (52). It has been reported that NF-κB played an important role in M1 macrophage polarization and was required for the expression of a many pro-inflammatory genes (53). Activated M1 macrophages expressed high levels of pro-inflammatory cytokines (IL-1β, IL-6, TNF-α, IL-12, IL-23, and Type-I IFN) and chemokines (CXCL1, 2, 3, 5, 9, 10) (54). CD14^+^ macrophages M1 were accumulated in the inflamed human intestine, enhancing antigen presentation and the subsequent inflammatory cascade. CXCL1 has been shown to mediate neutrophil recruitment by binding and activating CXCR2 expression on neutrophils during colitis in an M1-Toll-like receptor (TLR) signaling-dependent pathway (55). Animal studies have demonstrated that genetic deficiency for negative regulators of the canonical NF-κB pathway increase susceptibility to colonic inflammation (56, 57). In line with these findings, colitis was ameliorated by NF-κB decoy oligonucleotides targeting the consensus NF-κB binding site (58).

Our results indicated that *MMP1* and *MMP10* expression was significantly upregulated in the areas of ulcerated and inflamed intestinal mucosa. MMPs are a large family of zinc-dependent endoproteinases that degrade the extracellular matrix directly or indirectly by cleavage of protein substrates, such as TNF-α and IL-1β, thereby controlling various aspects of inflammation and immunity (59). IL-1β is a classic and potent pro-inflammatory cytokine, with elevated levels in UC tissue and intestinal mononuclear cells extracted from active UC specimens (60).

During intestinal inflammation, MMPs act as major effector molecules driving mucosal injury. MMP-1 expression reflects acute tissue injury and is associated with the initial steps of ulceration and angiogenesis in UC (61). In inflammatory bowel disease, MMP-1 activity is closely associated with myeloperoxidase (MPO) levels (62). MPO is an enzyme produced by neutrophils and released upon inflammatory stimulation, catalyzing the generation of highly cytotoxic and tissue-damaging reactive oxygen metabolites (63). Increased MPO levels are observed in the mucosa and intestinal lumen during inflammatory disease (64), promoting the inflammatory process (65). Inhibition of MMP-10 activity *via* siRNA or blocking associated signaling resulted in the amelioration of colitis. Recent findings indicated that increased levels of serum MMP-10 represent an early event in the pathogenesis of UC (66). MMPs are also known to influence macrophage behavior, potentially involving their antigen-presenting function.

DCs are heterogeneous cells that monitor the surrounding microenvironment and induce tolerance or incite a host defense pro-inflammatory response (67). Mucosal DCs exhibit unique properties that enable them to interact with T cells, B cells, the intestinal epithelium, and stroma, contributing to the maintenance of mucosal homeostasis or inducing inflammation (68). This dual function endows DCs with the capacity to bridge the innate and adaptive immunity. Mature DCs exhibit an upregulation of MHC II molecule, co-stimulatory molecule, and pro-inflammatory cytokine levels that enable the stimulation of pathogen-specific T cells (69). Innate sensing of microbes *via* distinct pathogen-recognition receptors enables DCs to launch distinct classes of T helper responses. DCs control Th1 responses through a mechanism of TLR signaling, involving MyD88- and TRAF6-mediated NF-κB activation (70). By contrast, some TLR2 ligands stimulate DCs to produce IL-10 or activate NOD1/NOD2 signaling to skew the balance toward Th2 responses (71, 72). Murine DC subsets in the intestine produce large amounts of IL-23, which plays critical roles in the induction of Th17 responses (73).

Identification of key miRNAs and lncRNAs involved in colitis pathogenesis provided new strategies for disease diagnosis and treatment. MiRNAs are endogenously expressed short nucleic acids, which regulate the expression of target mRNA *via* complementary sequences within the 3′-untranslated region, and can be orchestrated by lncRNAs. *In vivo* study demonstrated that upregulation of miR-129 ameliorated intestinal inflammation in TNBS-induced colitis mice through inhibition of the NF-κB signaling pathway (74). Lnc-ITSN1-2 was reported to function as a ceRNA to sponge miR-125a, thereby enhancing IL-23R expression and increasing disease risk, activity, and inflammatory cytokine profiles of IBD (75). In addition, lncRNA ANRIL accelerated UC development *via* the miR-323b-5p/TLR4/MyD88/NF-κB pathway (76). XIST regulated NF-κB/NLRP3 inflammasome pathway for mediating the process of inflammation (77). Taken together, the predicted lncRNA XIST and three key miRNAs may play important roles in the progression of UC by targeting the NF-κB pathway.

Intestinal injury arises through a programmed, coordinated series of events. Multiple immune cell types engage in inflammatory network and interact sequentially through cytokines and/or chemokines during the inflammatory process. The constructed ceRNA network potentially acted as an upstream regulator. CXCL1 and IL-1β were considered to mediate communication, and MMPs were shown to act as important effectors of the inflammatory process. Although our studies were detailed and comprehensive, the effective clinical information was lacking and requires clinical studies. Further research is warranted to narrow the gaps between basic, clinical, and translational application.

## Conclusion

Our study identified up-regulated expression of four hub genes and further revealed the action mechanisms of AGP in active UC. We also predicted the role of the XIST–miR-9-5p/miR-129-5p/miR-340-5p/NF-κB axis targeting NF-κB expression in active UC treatment. The ceRNA network obtained from the bioinformatics analysis and its potential function in combination with AGP can be examined in future experiments. Our findings may reflect the “tip of the iceberg” of the mechanism underlying inflammatory events that eventually cause colonic damage. Further advanced methods, such as single-cell sequencing technology, will provide new insights into disease mechanisms as well as novel therapeutic targets and open avenues for disease prediction and interventions.

## Data Availability Statement

The datasets presented in this study can be found in online repositories. The names of the repository/repositories and accession number(s) can be found in the article/**Supplementary Material**.

## Ethics Statement

The animal study was reviewed and approved by Ethics Committee of Soochow University.

## Author Contributions

MX and YZ designed, processed the data and completed the original draft manuscript; YK, NC and JL analyzed and organized the GEO datasets samples; WP, RZ and MJ reviewed and revised the manuscript; JZ, YW and JY constructed the colitis model; YC administrated the project and provided the funding. All authors contributed to the article and approved the submitted version.

## Funding

This work was supported by National Natural Science Foundation of China (No. 81820108023) and National Key Research and Development Program of China (No.2018YFC1705505); PHD Pre-research Funding of the Second Affiliated Hospital of Soochow University (No. SDFEYGJ2002) and Priority Academic Program Development of Jiangsu Higher Education Institutions.

## Conflict of Interest

The authors declare that the research was conducted in the absence of any commercial or financial relationships that could be construed as a potential conflict of interest.

## Publisher’s Note

All claims expressed in this article are solely those of the authors and do not necessarily represent those of their affiliated organizations, or those of the publisher, the editors and the reviewers. Any product that may be evaluated in this article, or claim that may be made by its manufacturer, is not guaranteed or endorsed by the publisher.
