# Supplementary figures and images for: Identification of Immune-Related Gene Signature and Prediction of CeRNA Network in Active Ulcerative Colitis
Source: Front Immunol. 2022 Mar 22;13:855645. doi: 10.3389/fimmu.2022.855645 (PMC8980722; doi:10.3389/fimmu.2022.855645)

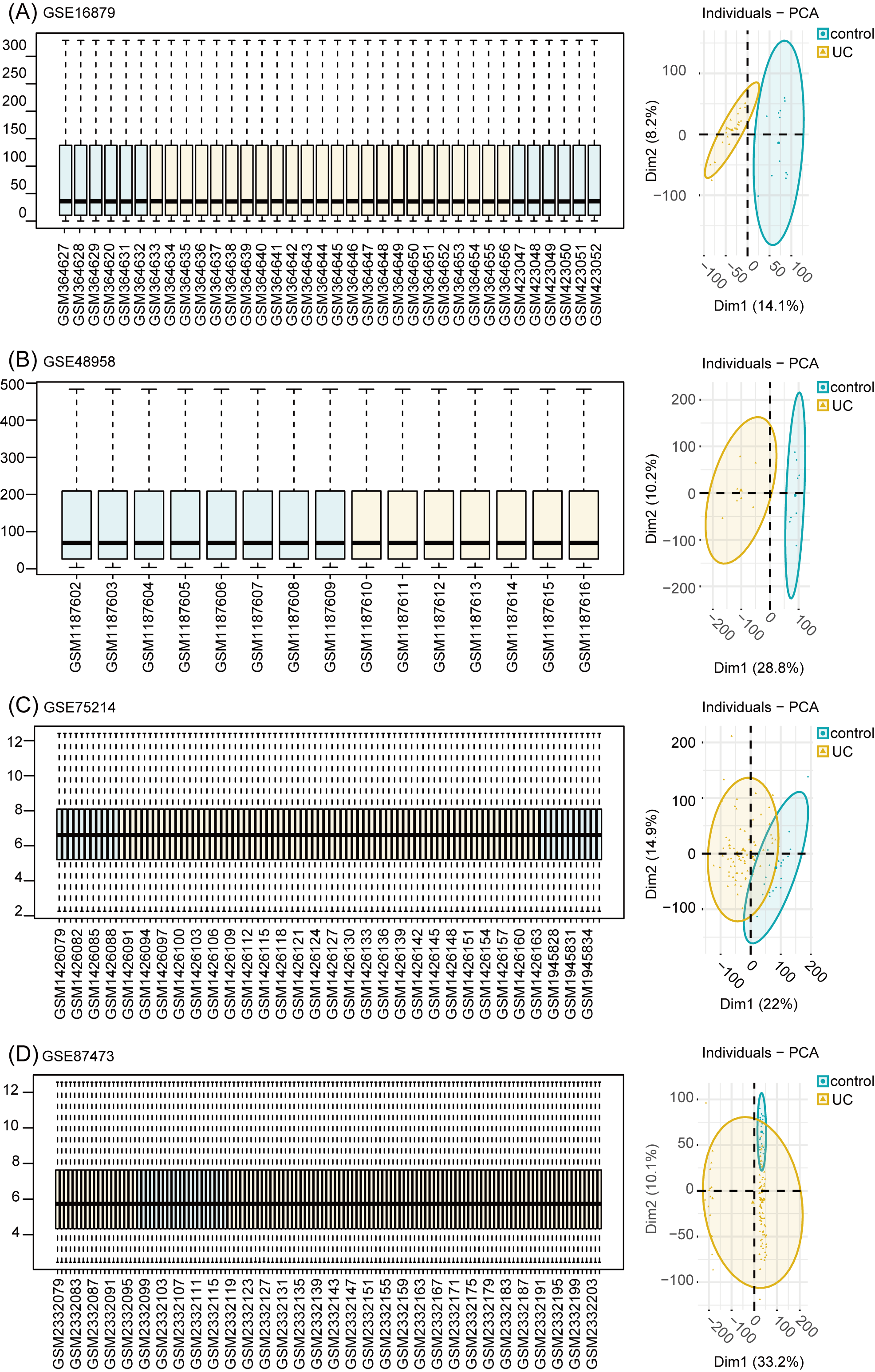

Supplement: Supplementary file 1 [file Image_1.tif]
